# Maternal Alcohol Consumption During Pregnancy and the Risk of Autism Spectrum Disorders in Offspring: A Retrospective Analysis of the Millennium Cohort Study

**DOI:** 10.1007/s10803-018-3626-6

**Published:** 2018-06-13

**Authors:** C. Gallagher, F. P. McCarthy, R. M. Ryan, A. S. Khashan

**Affiliations:** 1 School of Medicine, University College Cork, Cork, Ireland; 2 The Irish Centre for Fetal and Neonatal Translational Research, University College Cork, Wilton, Ireland; 3 Cork University Maternity Hospital, University College Cork, Cork, Ireland; 4 Department of Women and Children’s Health, School of Life Course Sciences, St Thomas’ Hospital, King’s College London, London, UK; 5 London School of Hygiene and Tropical Medicine, London, UK; 6 School of Public Health, University College Cork, Cork, Ireland

**Keywords:** Maternal alcohol consumption, Autism spectrum disorder, Pregnancy

## Abstract

The objective of this retrospective analysis of the longitudinal Millennium Cohort Study was to examine whether maternal alcohol consumption in pregnancy (MACP) is associated with the development of childhood autism spectrum disorders (ASD). Data on MACP and ASD were obtained from parental questionnaires. There were 18,168 singleton mother–child pairs with data on MACP, and 12,595 answered the question on ASD when the children were 11 years old. No statistically significant association was found between MACP and ASD for light (OR 0.78, 95% CI 0.48–1.29), moderate (OR 0.89, 95% CI 0.35–2.27), or heavy (OR 1.54, 95% CI 0.56–4.21) MACP. Alcohol consumption during pregnancy was not associated with the risk of developing ASD in this study cohort.

## Introduction

Autism spectrum disorders (ASD) are a group of common neuro-developmental disorders characterised by difficulties in social interaction, verbal and non-verbal communication, and repetitive behaviours (American Psychiatric 2013). The prevalence of ASD is approximately 1 in 130 globally and 1 in 68 in developed countries (Baxter et al. 2014; Boilson et al. 2016; Christensen et al. 2016). Worldwide, ASD is responsible for significant individual, caregiver and financial burden, with the overall national cost of ASD estimated to be £25 billion in the UK and $268 billion in the USA (Knapp et al. 2009; Leigh and Du 2015).

Whilst many causes of ASD have been proposed, most are thought to occur secondary to a combination of autism risk genes and environmental factors influencing early brain development. Genetic factors are believed to be responsible for approximately 50% of the risk, whilst shared environmental factors account for the remainder (Hallmayer 2011; Sandin et al. 2014). One potential risk factor is maternal alcohol consumption during pregnancy (MACP), which has been associated with numerous neurodevelopmental problems in children (Eliasen et al. 2010; Senturias 2014). Recent data indicates that MACP is a common occurrence in the western world with between 20 and 80% of mothers admitting to drinking alcohol, and up to 45% binge drinking during pregnancy (O’Keeffe et al. 2015).

The literature to date has largely focused on associations between fetal alcohol spectrum disorder (FASD) and ASD (Aronson et al. 2008; Landgren et al. 2010; Mukherjee et al. 2011). The extent of alcoholism in families of children diagnosed with ASD has also been investigated (Miles et al. 2003). To date, two case-control studies and two cohort studies have been conducted and did not find a relationship between MACP and ASD (Eliasen et al. 2010; Perrone-McGovern et al. 2015; Visser et al. 2012; Williams et al. 2003). Based on this limited current evidence, low to moderate alcohol consumption does not appear to be associated with the development of childhood ASD. However, there is insufficient evidence to draw any conclusions between high MACP and risk of ASD. This study was conducted to assess the association between low, moderate and high MACP and ASD risk.

## Methods

The Millennium Cohort Study (MCS) is an ongoing, nationally representative longitudinal study of 18,827 children born in the United Kingdom (UK) between 2000 and 2002. The design and conduct of the survey have been described in detail elsewhere (Connelly and Platt 2014). Briefly, 18,553 families agreed to participate in the first sweep of the survey, an interview response rate of 85%. Households were identified through the Department of Work and Pensions child benefit system and were selected based on where the family was resident shortly after the time of birth. All parents of children up to the age of 16 years are eligible to receive child benefit and coverage is estimated at 98%. The sample of births selected for the first survey of the MCS was clustered geographically, and disproportionately stratified to over-represent areas with high proportions of ethnic minorities in England, residents of areas of high child poverty and residents of the three smaller countries of the UK. Participants were not directly involved in this study, however there is ongoing research in patient engagement in the MCS as a whole (Wallace et al. 2013).

### Participants

There have currently been six main sweeps of data collection, involving home interviews and self-completion questionnaires, at ages 9 months and 3, 5, 7, 11 and 14 years. The majority of main respondents (99.7%) were the infant’s biological mother. A range of health-related data have been acquired as well as measures concerning child development, cognitive ability and educational attainment (Connelly and Platt 2014). When cohort members were 9 months old, mothers were asked whether they drank alcohol during pregnancy (every day; 5–6, 3–4, 1–2 days per week; 1–2 times per month; less than once per month; never). If the mother drank at least once or twice per week, she was asked: ‘In an average week, how many units of alcohol did you drink?’. If she drank once or twice per month or less than once per month, she was asked: ‘On the days when you did drink alcohol, on average how many units did you drink in a day?’. Mothers were told: ‘By a unit, I mean half a pint of beer, a glass of wine or a single measure of spirit or liqueur’. There are no globally accepted criteria on the levels of alcohol that constitute light, moderate or heavy drinking (Abel et al. 1998). In alignment with previous research into the effect of MACP in the MCS, we defined light and heavy drinking on the criteria outlined by the UK National Alcohol Strategy, which is consistent with the UK Department of Health guidelines for drinking during pregnancy (Department of Health, Home Office, Department for Education and Skills and Department for Culture, Media and Sport 2007; Kelly et al. 2008, 2013). Moderate drinking was defined as alcohol consumption at levels greater than light drinking, and less than heavy drinking. Drinking categories were defined as described in Table 1.

**Table 1:** Alcohol categories in pregnancy as defined in the MCS

None: subdivided into	(a) Never drinks alcohol (teetotal) (b) Drink alcohol but not during pregnancy
Light	Not more than 1–2 units per week or at any one time in pregnancy
Moderate	Not more than 3–6 units per week or 3–5 units at any one time in pregnancy
Heavy	7 or more units per week or 6 or more units at any one time in pregnancy

In sweep five, when cohort members were 11 years of age, the main respondent was asked if a doctor or health professional had ever told them that their child had Autism, Asperger’s syndrome or other ASD. The outcome measure of ASD was based on a positive or negative response to this question.

Potential confounding variables and mediators were determined a priori based on previous literature. Data on variables were extracted from the primary survey when infants were 9 months of age. These include maternal age at interview, paternal age at interview, maternal marital status, ethnicity, education, household income, social deprivation, depression and depression medication at time of interview, maternal body mass index (BMI) immediately before pregnancy, maternal smoking status during pregnancy, maternal hypertension during pregnancy, diabetes before or during pregnancy, infant sex, gestational age, birthweight and mode of delivery.

### Data Analysis

Baseline characteristics of all mother–child pairs are summarised based on maternal drinking category (Table 2). Crude (model A) and adjusted logistic regression was used to estimate the association between MACP and the ASD. Potential confounders were subdivided into three categories; parental age, socio-demographic factors and maternal health factors (Table 3). Each category of variables was modelled separately in adjusted analysis (models B–D). A final model (model E) was run using the four factors which were likely to be potential confounders in the previous adjusted models; household income, ethnicity, maternal smoking and BMI. For these models, the ‘none’ drinking category which includes non-drinker women and drinkers who stopped drinking before in early pregnancy was used as the reference group.

**Table 2:** Maternal and neonatal characteristics according to maternal alcohol consumption during pregnancy

	Categories of maternal alcohol consumption during pregnancy			
	None (%)	Light (%)	Moderate (%)	Heavy (%)
Total (%)	67.3	25.2	5.4	2.1
Maternal age at first interview (years)				
13–19	5.4	2.9	5.8	7.6
20–24	18.1	10.5	13.5	22.3
25–29	26.3	21.7	20.6	19.7
30–34	31.4	38.9	32.1	25.4
35–39	15.9	21.6	22.6	17.2
40+	3.0	4.4	5.4	7.9
Ethnicity				
White/Caucasian	85.2	96.0	97.7	97.3
Asian	9.5	1.0	0.3	0.3
African/Afro-Caribbean	3.0	1.6	0.7	0.6
Other	2.2	1.4	1.3	1.8
Maternal education				
Higher degree	2.9	6.0	4.4	3.9
First degree	11.4	22.6	18.1	12.3
Diplomas in higher education	8.9	11.3	9.2	11.6
A/AS/S levels	9.4	11.4	8.2	7.5
O level/GCSE grades A–C	35.9	32.1	32.8	32.4
GCSE grades D–G	11.7	8.2	9.8	10.6
Other/overseas	2.9	1.0	0.7	0.3
None	16.9	7.4	16.7	21.4
Marital status of main responder				
Legally separated	2.6	1.9	3.1	0.9
Married, 1st and only marriage	56.5	62.1	49.5	34.5
Remarried, 2nd or later marriage	4.6	4.7	4.3	5.7
Single never married	31.8	27.0	37.1	53.4
Divorced	4.4	4.2	5.7	5.0
Widowed	0.2	0.1	0.3	0.6
Household income				
£0 to £3099	1.6	1.0	0.4	3.4
£3100 to £10,399	22.9	12.6	19.9	27.4
£10,400 to £20,799	33.6	26.6	28.3	30.9
£20,800 to £31,199	21.9	24.1	19.4	17.8
£31,200 to £51,999	15.0	23.8	20.2	12.7
£52,000 and above	5.0	11.9	11.8	7.8
Social deprivation (decile)				
min-9 (most deprived)	13.5	5.6	12.3	13.8
10–19	11.3	7.5	10.1	14.0
20–29	11.4	7.6	10.6	15.6
30–39	9.3	8.9	6.8	7.3
40–49	10.6	9.2	9.6	9.3
50–59	9.4	10.9	10.6	10.5
60–69	8.9	11.0	8.0!	7.4
70–79	8.6	12.3	10.5	8.6
80–89	8.5	12.5	10.4	7.8
90-max (least deprived)	8.5	14.7	11.2	5.7
Maternal BMI				
BMI < 18.5	6.1	3.6	4.7	8.1
BMI 18.5–24.9	64.0	71.1	71.3	64.8
BMI 25.0–29.9	20.1	19.2	17.1	21.1
BMI 30+	9.8	6.2	6.9	6.0
Maternal smoking during pregnancy				
Non-smoker^a^	68.8	69.2	58.3	39.8
Gave up	15.5	18.0	19.4	27.0
Smoker	15.6	12.9	22.4	33.1
Gestational hypertension/preeclampsia/eclampsia				
Yes	8.2	6.1	5.5	7.4
Maternal diabetes				
Yes	2.1	1.3	1.0	1.4
Maternal depression				
Yes	24.0	23.2	26.1	32.6
Paternal age				
13–19	0.9	0.5	0.5	2.3
20–24	8.6	4.8	5.9	10.5
25–29	20.1	15.6	13.2	17.4
30–34	34.7	34.3	32.2	25.2
35–39	24.1	30.3	30.6	25.2
40+	11.7	14.5	17.7	19.4
Infant sex				
Male	51.0	51.7	53.2	54.2
Gestational age				
24–36 weeks	7.2	5.9	5.4	7.1
37 weeks	5.9	4.7	5.3	6.3
38 weeks	14.1	12.6	13.7	13.0
39 weeks	20.6	22.7	19.7	21.8
40 weeks	28.6	28.0	29.4	25.9
41+ weeks	23.7	26.0	26.5	26.0
Infant birthweight				
0–1.49 kg	1.0	0.5	0.1	1.1
1.5–2.49 kg	5.5	4.3	6.1	6.3
2.5–3.99 kg	81.1	81.3	81.7	81.1
4+ kg	12.4	13.9	12.1	11.5

^a^Non-smokers defined as women who have not smoked in previous 2 years from date of first

**Table 3:** The potential variables included in each logistic regression model

Model	Category of variable	Variables
A	Crude analysis	N/A
B	Parental age	Maternal age, paternal age
C	Socioeconomic/demographic factors	Household income, maternal education, social deprivation, ethnicity, marital status
D	Maternal health factors	Maternal smoking, BMI, hypertension, diabetes, depression treatment
E	Combined	Household income, ethnicity, maternal smoking, BMI

Furthermore, models A–E were repeated using an alternative categorisation of MACP (which divided the ‘none’ drinking category into ‘drinks but not during pregnancy’ and ‘teetotal’ groups). Initially the ‘drinks but not during pregnancy’ was used as the reference group as, from a public health point of view it is important to assess the impact of continuing drinking compared to stopping drinking. This analysis was repeated using the ‘teetotal’ group as reference, to reduce the confounding of epigenetic changes related to pre-pregnancy alcohol consumption altering the risk for having a child with ASD (Appendix 1). Subgroup analysis was carried out with the original alcohol classification system using model E including only babies who were male sex, White ethnicity or firstborn (Appendix 2). Results are reported as odds ratios (ORs) and 95% CIs. As pregnancy outcome measures (gestational age, birthweight and mode of delivery) were more likely to be mediating factors, these were not included in the main analysis but tested separately and individually, with each being added onto model E.

Survey commands were used and estimates were weighted to account for the complex survey design. Original survey design weights were used as it has been previously shown that these are not meaningfully different from the subsequent combined and loss to follow-up weights (Plewis 2004). All data analysis was carried out using the statistical software package SPSS (IBM v.23).

## Results

There were 18,168 singleton mother–child pairs with data on MACP from sweep 1 of data collection. Of these 12,595 answered the question on ASD in sweep 5, resulting in 205 (1.6%) reported cases of ASD. Of these cases 146 (71.2%) were in the none, 44 (21.4%) in the light, 9 (4.4%) in the moderate and 6 (3%) in the heavy alcohol category respectively.

### Patterns of MACP

12,125 cohort members (67.3%) reported not consuming any alcohol during pregnancy, with 4544 (25.2%) reporting light, 974 (5.4%) moderate and 374 (2.1%) heavy consumption respectively. Several patterns emerged within the different drinking categories (Table 2). Light and moderate drinkers tended to be older, better educated, married, have a normal BMI and higher total household incomes compared to the other groups. Heavy drinkers on the other hand, tended to be the youngest, unmarried, smoke during pregnancy and come from low income households. The ‘none’ drinking category had a significantly bigger proportion of non-White cohort members (14.8%) compared to all the other groups (2.3–4%). Furthermore, they had the lowest levels of maternal education, were the least likely to be smokers, and like the heavy drinking group, tended to be younger and come from lower income families. Of those who dropped out of the study, 71.6% reported never drinking and 2.0% reported heavy drinking during pregnancy. These figures were similar to those who remained in the study, of whom 65.4% reported never drinking, and 2.1% reported heavy drinking (Appendix 3).

### Logistic Regression Results

No statistically significant association was found between MACP and development of ASD in crude and adjusted analysis (Table 4). In the crude analysis, there was a negative but statistically non-significant association between light (OR = 0.72; [95% CI 0.48–1.08]) and to a lesser extent moderate (OR = 0.78; [95% CI 0.33–1.87]) MACP compared to not drinking during pregnancy and the development of ASD. Among heavy drinkers, the OR of having a child with ASD was increased compared to non-drinkers (OR = 1.53; [95% CI 0.67–3.5]), however this result was not statistically significant. This trend for light, moderate and heavy MACP was consistent in all subsequent adjusted analyses with no statistically significant associations. When the second classification system of MACP was used, the results followed a similar pattern to above, regardless of whether the ‘drinks but not during pregnancy’ or ‘teetotal’ group were used as reference. These results are presented in Appendix 1.

**Table 4:** The association between maternal alcohol consumption during pregnancy and the risk of autistic spectrum disorders

MACP	ASD cases	Model A OR (95% CI)	Model B OR (95% CI)	Model C OR (95% CI)	Model D OR (95% CI)	Model E OR (95% CI)
None^a^	146	Ref.	Ref.	Ref.	Ref.	Ref.
Light	44	0.72 (0.48, 1.08)	0.72 (0.44, 1.16)	0.8 (0.51, 1.26)	0.72 (0.45, 1.15)	0.78 (0.48, 1.29)
Moderate	9	0.78 (0.33, 1.87)	0.94 (0.34, 2.55)	0.8 (0.31, 2.08)	0.8 (0.31, 2.04)	0.89 (0.35, 2.27)
Heavy	6	1.20 (0.47,3.05)	1.55 (0.55, 4.4)	1.15 (0.44, 2.97)	1.43 (0.54, 3.82)	1.54 (0.56, 4.21)

Model A (crude analysis)

Model B (parental age): maternal age, paternal age

Model C (socioeconomic/demographic factors): household income, maternal education, social deprivation, ethnicity, marital status

Model D (maternal health factors): maternal smoking, BMI, hypertension, diabetes, depression treatment

Model E (combined): household income, ethnicity, maternal smoking, BMI

^a^The none category include non-drinker women and drinkers who stopped drinking before/in early pregnancy

Adjustment for pregnancy outcome measures (gestational age, birthweight and mode of delivery) in separate models did not change the results materially. Confining the analysis to White/Caucasian ethnicity, male offspring sex or primiparous women did not change the association between MACP and ASD using the original alcohol categories. In these subgroup analyses, the number of exposed cases in the moderate and high MACP categories were very small and the results were not meaningful. These results are presented in Appendix 2.

## Discussion

### Main Findings

In this large retrospective population-based cohort study, the role of MACP during pregnancy on development of ASD was investigated. We found no significant associations between MACP and ASD in the overall study cohort. In our study, there was a non-significant trend for light and moderate alcohol consumption to reduce risk of ASD compared to the ‘none’ drinking group. Contrastingly, heavy alcohol consumption appeared to increase risk for ASD, but this result did not reach statistical significance. The fact that adjustment for pregnancy outcome measures (gestational age, birthweight and mode of delivery) did not change the results suggests that they do not play a significant confounding or mediating role in the association between MACP and the risk of ASD.

### Strengths and Limitations

The present study has several strengths. It was based on a large, nationally representative contemporary cohort of children born in the UK. The very high response rate (85%) for the first survey data compared to the only other large cohort study (30%), means that selection bias is less likely in our study (Landgren et al. 2010). Furthermore, there were no significant differences in alcohol consumption between those who remained in the study, and those who dropped out of the study prior to ASD data collection (Appendix 3). Our dataset contains information on a wide range of potential confounding factors, including some which have been unavailable in previous investigations but have been shown to influence the risk for ASD (Leonard et al. 2011; Lyall et al. 2010; Zaroff and Uhm 2011). Finally, this is the first article to our knowledge to subdivide women who did not drink during pregnancy into those who never drink and those who drink but gave up during pregnancy. The never drinking group displayed several distinct trends in important potential confounding variables (ethnicity, household income, BMI). Notably, the ‘never drinking’ group had a significantly different ethnic make-up compared to all other groups with 59% of its population being White/Caucasian compared to 95% in the ‘drank but gave up during pregnancy’ group. This suggests that this unique subpopulation carry a risk profile different from other alcohol consumption groups, and perhaps that the second alcohol categorising system is more appropriate when analysing risk factors for ASD.

This study has several limitations. The low number of ASD cases, especially in the moderate and high alcohol consumption categories (nine and six respectively) leads to limitations in statistical power. This could possibly explain previous research indicating increased risk for ASD among heavy drinking populations such as alcoholic mothers, and mothers of children with FASD (Aronson et al. 2008; Landgren et al. 2010; Mukherjee et al. 2011; Stockwell et al. 2004). Future research investigating the effect of moderate/high levels of MACP on risk of ASD should ensure larger sample sizes in order to guarantee sufficient ASD cases to carry out accurate analysis.

No information about the time-period of drinking during pregnancy is present in the MCS data, making any time dependent effects of alcohol on subsequent development of ASD impossible to investigate. No information regarding more refined drinking related variables (age of onset of drinking, prior drinking habits, diagnosis/treatment of alcohol use disorder etc.) is present in the MCS data. Such data may provide more clarity regarding MACP levels and possibly aid interpretation of results.

The exposure data were collected retrospectively. This raises potential issues with differential-recall and social desirability bias, especially in mothers of children displaying autistic symptoms. However, whilst a reliable diagnosis of ASD can be made as early as 2 years of age by an experienced clinician, most children don’t show recognisable symptoms before 12 months (Johnson and Myers 2007; Lord et al. 2006). Therefore the likelihood that a child would have been displaying symptoms of autism at the time of the first survey, and thus the likelihood of differential-recall bias was deemed to be low.

There is significant social stigma associated with drinking, especially during pregnancy with pregnant women frequently under-reporting alcohol consumption (Johnson and Myers 2007). Evidence on the reliability of prospective verses retrospective collection of data on alcohol consumption is mixed with some studies showing prospective studies provide the most accurate estimates, whilst others report that retrospective studies are more reliable (Alvik et al. 2006; Corti et al. 1990). In our study 32.7% of women reported drinking alcohol at some point during pregnancy, this is reasonably consistent with other studies in western countries, implying that the data collected on MACP is reasonably reliable (Abel et al. 1998; O’Keeffe et al. 2015).

The question investigating ASD—“has a doctor or health professional ever told you that your child has ASD”—may have led to misreporting of cases. Data on the validity of parentally reported neurodevelopmental disorders is lacking, though surveys including parental reported ASD have been used in previous research studies (Russell et al. 2013). It should be noted that prevalence of ASD in the study cohort is similar to recent estimates in other developed countries, reducing the likelihood of misreporting (Christensen et al. 2016).

Finally, as with all cohort studies, loss to follow up is a potential limitation of the current study. Whilst the original study cohort consisted of 18,168 mother child pairs, by sweep five, when data on ASD was collected only 12,595 responded. However it should be noted that previous studies have shown that the original sample weights at 9 months of age, which were used in this analysis, appeared to still be valid at 7 years (Plewis 2004). Furthermore, as shown in Appendix 3, there were no differences in MACP between those who completed follow up to age 11 and those who dropped out of the study prior, and so there was no differential loss to follow up in respect of outcome in this cohort.

### Interpretation

The evidence to date regarding the association between MACP and ASD has been conflicting. Two case-control studies have been conducted and did not find a relationship between MACP and ASD. In fact, both reported a significantly lower occurrence of MACP in cases of ASD compared to matched controls, suggesting MACP was protective against ASD (Visser et al. 2012; Williams et al. 2003). Data from two cohort studies examining the relationship between MACP and ASD is currently available (Landgren et al. 2010; Perrone-McGovern et al. 2015). This includes a large well designed prospective study including 80,552 children (Landgren et al. 2010). This study found no association between MACP, number, or timing of binge drinking episodes and childhood ASD, and presented reliable data showing that low to moderate alcohol consumption during pregnancy is not associated with development of ASD. The results of our study are in line with this.

In univariate analysis, we found that ethnicity, income, maternal BMI and maternal smoking during pregnancy, had the greatest effect on the association between MACP and ASD of all included covariates. Thus it seems prudent that future research should include these factors in their analysis.

## Conclusion

In conclusion, light moderate or heavy alcohol consumption during pregnancy was not associated with the risk of developing ASD in this study cohort. Due to the limited number of cases, results were limited by statistical power and more research is warranted to investigate the relationship between heavy MACP and ASD. Future studies are needed to investigate the effects of antenatal binge drinking and the gestational timing of such episodes on development ASD.

## Appendix 1

See Tables 5 and 6.

**Table 5:** Logistic regression analysis, using ‘no alcohol’ category subdivisions

MACP	ASD cases	Model A	Model B	Model C	Model D	Model E
Drinks alcohol but gave up during pregnancy	112	Ref.	Ref.	Ref.	Ref.	Ref.
Never drinks alcohol (teetotal)	34	0.88 (0.58, 1.33)	0.86 (0.53, 1.39)	1.05 (0.68, 1.63)	0.87 (0.52, 1.44)	1 (0.58, 1.73)
Light	44	0.7 (0.46, 1.06)	0.69 (0.42, 1.13)	0.81 (0.52, 1.27)	0.69 (0.43, 1.13)	0.78 (0.47, 1.29)
Moderate	9	0.76 (0.31, 1.83)	0.9 (0.33, 2.5)	0.81 (0.31, 2.1)	0.77 (0.3, 1.98)	0.89 (0.35, 2.26)
Heavy	6	1.16 (0.46, 2.96)	1.49 (0.53, 4.22)	1.16 (0.45, 3.01)	1.39 (0.52, 3.72)	1.54 (0.56, 4.24)

“Drinks alcohol but gave up during pregnancy” as reference group

Model A (crude analysis)

Model B (parental age): maternal age, paternal age

Model C (socioeconomic/demographic factors): household income, maternal education, social deprivation, ethnicity, marital status

Model D (maternal health factors): maternal smoking, BMI, hypertension, diabetes, depression treatment

Model E (combined): household income, ethnicity, maternal smoking, BMI

**Table 6:** Logistic regression analysis, using ‘no alcohol’ category subdivisions

MACP	ASD cases	Model A	Model B	Model C	Model D	Model E
Never drinks alcohol (teetotal)	34	Ref.	Ref.	Ref.	Ref.	Ref.
Drinks alcohol but gave up during pregnancy	112	1.14 (0.75, 1.74)	1.17 (0.72, 1.89)	0.95 (0.61, 1.48)	1.16 (0.7, 1.92)	1 (0.58, 1.74)
Light	44	0.79 (0.47, 1.34)	0.81 (0.44, 1.49)	0.77 (0.43, 1.38)	0.8 (0.43, 1.48)	0.79 (0.4, 1.56)
Moderate	9	0.86 (0.35, 2.14)	1.05 (0.37, 2.96)	0.77 (0.28, 2.13)	0.89 (0.32, 2.51)	0.89 (0.31, 2.6)
Heavy	6	1.33 (0.49, 3.6)	1.74 (0.56, 5.45)	1.1 (0.4, 3.06)	1.6 (0.56, 4.61)	1.55 (0.52, 4.59)

“Never drinks alcohol (teetotal)” group as reference group

Model A (crude analysis)

Model B (parental age): maternal age, paternal age

Model C (socioeconomic/demographic factors): household income, maternal education, social deprivation, ethnicity, marital status

Model D (maternal health factors): maternal smoking, BMI, hypertension, diabetes, depression treatment

Model E (combined): household income, ethnicity, maternal smoking, BMI

## Appendix 2

See Table 7.

**Table 7:** Subgroup analysis, restricted by ethnicity, infant sex and maternal parity

MACP	Model E OR (95% CI)	White ethnicity only	Male infants only	Primiparous women only
None^a^	Ref.	Ref.	Ref.	Ref.
Light	0.78 (0.48, 1.29)	0.77 (0.45, 1.3)	0.88 (0.5, 1.56)	0.42 (0.16, 1.1)
Moderate	0.89 (0.35, 2.27)	0.78 (0.3, 2.02)	0.79 (0.25, 2.44)	0.47 (0.09, 2.36)
Heavy	1.54 (0.56, 4.21)	1.6 (0.58, 4.4)	1.48 (0.4, 5.44)	3.54 (0.77, 16.38)

Model E (combined): household income, ethnicity, maternal smoking, BMI

^a^The none category include non-drinker women and drinkers who stopped drinking before/in early pregnancy

## Appendix 3

See Table 8.

**Table 8:** Comparison of those who dropped out of study versus those who followed up, by MACP

MACP	Remained in the study (%)	Dropped out (%)
None	65.4	71.6
Light	27.1	20.9
Moderate	5.4	5.5
Heavy	2.1	2.0
